# Challenges in Patients with Trisomy 21: A Review of Current Knowledge and Recommendations

**DOI:** 10.1155/2021/8870680

**Published:** 2021-05-26

**Authors:** Jennifer Robinson, Manca Tekavčič Pompe, Christina Gerth-Kahlert

**Affiliations:** ^1^Department of Ophthalmology, University Hospital Zurich, University of Zurich, Zurich, Switzerland; ^2^University Eye Clinic, Ljubljana, Slovenia

## Abstract

**Purpose:**

To summarize and review the common ophthalmic anomalies in children with trisomy 21 (Down syndrome) in order to propose an update to current clinical recommendations.

**Methods:**

A retrospective chart review, systemic literature review, and international survey of the frequency of ocular abnormalities, screening schedules, and challenging aspects examining children with trisomy 21. The chart review included patients treated at the Department of Ophthalmology at the University Hospital of Zurich over a two-year period. The international survey was submitted to the members of the Swiss Society of Ophthalmology, Slovenian Ophthalmological Society, and European Pediatric Ophthalmology Society.

**Results:**

Analysis of 52 patient records during the study period revealed refractive errors (astigmatism: 54% of patients, hyperopia: 26%, and myopia: 15%) as the most common diagnosis, whereas childhood cataract was reported in 5%. This is in concordance with the extended literature review of 249 publications, although congenital cataracts were reported to be higher than at our institution. The survey participants reported great challenges in taking care of these patients, despite their long professional experience (73% with over 10 years of experience).

**Conclusion:**

Care and treatment of children with trisomy 21 continues to be demanding for paediatric ophthalmologists. We recommend the following examination schedule for these patients: first, ophthalmological examination at 6–12 months of age, then once in 3–6 months for children under 2 years of age, once in 6 months for children 2–5 years of age, annually for children 5–10 years of age, and thereafter, to be decided on an individual basis depending on the presenting ocular abnormalities of the patient.

## 1. Introduction

Trisomy 21 is the most common chromosomal aberration, caused by trisomy of chromosome 21 in the majority of patients. Translocation of a third chromosome 21 or mosaicism is identified in less than 10% of patients. This syndrome is among the chromosomal defects with the highest awareness in public and health institutions. Trisomy 21 is, by definition, a rare/orphan disease. According to the FDA (U.S. Food and Drug Administration), an orphan disease is defined as affecting less than 200,000 people nationwide. In Switzerland, the proportion of live born babies with trisomy 21 was reported as 0.8‰ in 2016 (source: Bundesamt für Statistik). Typically, patients with trisomy 21 present with various levels of cognitive/intellectual disabilities and distinct physical features, and due to the various comorbidities associated with the condition, these patients require special care and, when necessary, treatment.

Very little is known regarding the distinct phenotype and characteristic facial dysmorphology in patients with trisomy 21. It is hypothesized that the chromosomal imbalance causes an increase of developmental instability which contributes to these characteristic features [1]. The visual system is affected in the majority of patients with trisomy 21. The most frequently reported ocular abnormalities amongst others are hyperopia [2, 3], astigmatism [3, 4], strabismus [5, 6], myopia [7, 8], and congenital cataract [9, 10], which underlines the importance of guidelines or recommendations in order to ensure the best possible treatment and early recognition of ocular pathologies in this vulnerable group of patients.

To date, few guidelines regarding the ophthalmic care of patients with trisomy 21 are available. In Europe, recommendations for recognizing and treating patients with trisomy 21 include (1) Nottingham Guidelines for the Management of Children with Down Syndrome and (2) eye tests for children with Down syndrome, published by Cardiff University. The American Association for Pediatric Ophthalmology and Strabismus recommends the first ophthalmologic examination of children with trisomy 21 at the age of 6 months and, thereafter, annually or more frequently if needed (https://aapos.org/glossary/down-syndrome). The aim of this paper is to review the current knowledge of common ophthalmic anomalies in patients with trisomy 21, to draw a comparison to the patient cohort treated at our institution, and to propose an update to current recommendations for the international community.

## 2. Methods

We conducted a retrospective chart review of patients with trisomy 21 treated at the Department of Ophthalmology at the University Hospital of Zurich over a two-year period (April 2017 to March 2019). The following data were analyzed from the clinical charts: age, follow-up time, type of examination (orthoptic/general ophthalmologic exam), best-corrected visual acuity, refractive errors, type of spectacles prescribed, intraocular pressure, anterior segment diseases (cataract and keratoconus), retinal or optic nerve findings, and presence or absence of strabismus. Clinical data were compared with published data regarding the most common ophthalmic pathologies in children with trisomy 21, extracted using a PubMed, Google Scholar, and Medline keyword search using the following search terms: children, ophthalmic, Down syndrome, and trisomy 21. We considered publications with details regarding the occurrence of ophthalmic anomalies or pathologies and have listed these in Table 1. Publications that did not list the incidence of ophthalmologic pathologies and anomalies in a tabular manner as well as such using outdated or derogatory terminology were excluded.

In order to reinforce our clinical analysis and literature review, we performed a survey to investigate current care practices in children with trisomy 21. The survey was sent to the members of three different organizations: the Swiss Society of Ophthalmology, Slovenian Ophthalmological Society, and European Pediatric Ophthalmological Society. The survey included thirteen queries and two open questions as listed in Table 1. The chart review and survey were approved by the Cantonal Ethics Committee of Zurich (BASEC No. 2019-00627) and required general consent by the patients' guardians.

## 3. Results

### 3.1. Retrospective Chart Review

During the study period, a total of 58 patients with trisomy 21 were identified. The majority of these patients were Caucasians of middle Europe. Permission to review their data was granted by the guardians of 52 patients (33 males, 19 females). The majority of patients were referred to our clinic by a paediatrician (60%), with the remainder referred by a primary-care physician (27%) or ophthalmologist (13%). Most patients received an orthoptic examination in addition to regular ophthalmological examinations. Age-adjusted best-corrected visual acuity was normal in 28% of the patients, whereas the remaining 72% presented with refractive disorders. High astigmatism of 2 dpt or more was the most common refractive error (54%), followed by hyperopia of more than +5 dpt (26%) and myopia of more than −6 dpt (15%). The level of hyperopia ranged from +0.5 dpt to +5 dpt, and the amount of myopia from −0.25 dpt to −6 dpt, whereas the level of astigmatism was documented from −0.5 dpt to −4 dpt. Nystagmus was present in 19% of patients and manifest strabismus in 38% (of which 70% was convergent and 30% was divergent). Cataract and keratoconus were the most common anterior segment diseases, documented in 5% and 6% of patients, respectively. Follow-up examinations were scheduled every 3 to 12 months, depending on the patient's age, visual development, and ocular disease. Treating ophthalmologists mainly prescribed monofocal spectacles (54%). Bifocals were prescribed in 26%, whereas in the remaining 20%, no correction was required as summarized in Table 2.

### 3.2. PubMed, Google Scholar, and Medbase Review

We identified 249 PubMed, 72 Google Scholar, and 219 Medbase publications published between 1980 and 2019 focusing on various ophthalmic anomalies in children with trisomy 21 using the keywords Down syndrome, trisomy 21, ophthalmic, and ophthalmologic. The incidence of ophthalmic pathologies varies according to the study, as summarized in Table 3. The most common ocular diseases were identified and analyzed with respect to the number of patients affected. Though findings varied between the respective studies, refractive errors (particularly astigmatism and hyperopia) were the most prevalent ophthalmic finding. Congenital cataract, congenital nasolacrimal duct obstruction, retinal anomalies, and glaucoma were listed less frequently.

### 3.3. Survey

The survey was completed by 147 participants after it had been sent to a total of 1057 ophthalmologists. Upon beginning the online survey, each participant agreed on the results being published. Participants were given the opportunity to decline before starting the survey, which none of them made use of. The detailed survey results can be found in Table S1 (supplementary material). The majority of participants (73.4%) were board-certified ophthalmologists with over 10 years of practical experience who were mainly from European countries other than Switzerland (Slovenia, Hungary, Sweden, Portugal, Belgium, UK, Spain, Poland, the Netherlands, Latvia, France, Finland, Austria, and Denmark). Board-certified ophthalmologists without a subspecialty represented 42% of all participants, while 37.4% were specialized in paediatric ophthalmology. Other subspecialties included anterior segment (22.9%), glaucoma (10.7%), retina (9.9%), neuroophthalmology (9.2%), oculoplastics (7.6%), and others (7.6%). The responding ophthalmologists predominantly worked in private practice (49.6%), followed by those working in a tertiary-care center (35.9%) and 22.9% employed in a secondary-care center. Data from survey participants who had never treated patients with trisomy 21 (11.3%) were excluded from the evaluation. The vast majority of responding ophthalmologists treated patients with trisomy 21 one to two times per month (78.4%), with a minority of respondents examining such patients more than ten times a month (4.9%). The mean patient age at which the first examination was performed varied between birth and 30 years of age. Almost half (45.6%) of the respondents did not adhere to a screening protocol when caring for patients with trisomy 21 without ophthalmic pathology. However, a total of 24.7% would like to make use of such protocols but are not aware of any. Patients were not frequently referred to low-vision specialists. Those referrals were always or regularly made by 6.8% and 17.5% of respondents, respectively, with 45.6% referring rarely and 30.1% never. The majority of survey participants (61.7%) had prescribed glasses with a near addition to their patients with trisomy 21. The greatest challenges in care and treatment of patients with trisomy 21 were reported as difficulties to examine the patients due to their reduced capacity to cooperate; difficulty to elicit a patient's history and evaluate their complaints; extra time needed to examine these patients; necessity of sedation during the examination; and reduced patient acceptance of the prescribed glasses. 88% of survey respondents agreed with the suggestion that adequate care in their respective country is provided to patients with trisomy 21.

## 4. Discussion

Results of our data analysis, literature review, and international survey corroborate that patients with trisomy 21 are prone to a variety of ocular pathologies and that special attention, care, and skills are required for taking care of this patient group.

Our retrospective analysis confirms astigmatism as the prevailing refractive error as similarly described in previous studies [1–9, 11–13]. Strabismus was recorded in more than one-third of our analyzed cohort; however, it is reported of variable frequency in the literature ranging from 22 to 57% as listed in Table 3. Cataract was documented in 5% of our patients, which is lower than the reported frequency of congenital cataract in patients with trisomy 21 of 10% or higher [2, 3, 5–7, 9–11]. This may reflect a referral bias in different institutions rather than a “true” incidence of 10%. Retinal anomalies were present in a small group of examined patients. In this study, we did not further investigate these retinal findings. However, Ugurlu and Altinkurt have found that individuals with trisomy 21 on average present with a higher central retinal thickness as well as higher retinal nerve fibre layer of the papilla [14]. Mangalesh et al. showed that already infants with trisomy 21 can present with retinal abnormalities such as abnormal foveal morphology [15].

It has also been shown that children with trisomy 21 tend to have significant accommodative deficiencies even if preexisting hyperopia has been corrected or if no refractive error has been measured [16]. The ethology is not fully clear. It is assumed that neurological or psychological deficiencies might be partially responsible [17]. These findings are crucial as vision plays an important role in childhood development. Accommodation ability in patients with trisomy 21 can be assessed even in unsettled children by dynamic retinoscopy with a streak retinoscope. The child is asked to fixate a near point target. The object is moved closer and further away from the child to seek for the neutral point at near which provides the accommodation response [18, 19].

It is important to prescribe the full cycloplegic retinoscope measures in patients with insufficient accommodation. Also, hyperopia, even if mild, should be fully corrected without any near subtractions.

To optimise near-vision we recommend prescribing bifocals with the bifocal segment top placed at the pupillary center [20]. This way, children with trisomy 21 can benefit from a significant improvement of near-vision in comparison to monofocal spectacles [19]. In addition, the visual and verbal learning ability can, thereby, be enhanced [21]. Bifocals are generally well tolerated by children with trisomy 21 [22].

The participants of our conducted survey stated that the most challenging part of caring for patients with trisomy 21 is performing a correct refraction due to a lack of cooperation. This is a crucial obstacle considering the fact that refractive errors are a common ocular pathology amongst this particular demographic [2–14, 16, 23–26] and that precise refractive correction has an impact on visual development.

The strength of this study is its documentation of the widespread difficulties encountered amongst physicians caring for patients with trisomy 21. These obstacles seemed to be similar across Europe, independent of the country of practice. Our aim was to illuminate this difficult subject, thus facilitating care and treatment of patients with trisomy 21. We hope that this study inspires a dialogue with the goal of establishing global recommendations for screening examinations in patients with trisomy 21.

This study has a potential limitation. Unfortunately, the survey response rate was not sufficiently high among members of the Swiss Society of Ophthalmology and European Pediatric Ophthalmological Society to draw a detailed international comparison and, hereby, suggest country-specific guidelines. However, the lack of recommendations in their respective countries of practice was apparent to many participants.

In conclusion, based on our data analysis, clinical experience, literature review, and the preexisting international recommendations, we propose the following protocol: first, ophthalmological and orthoptic examination between 6–12 months of age, followed by examinations every 3–6 months for children under 2 years of age (depending on their visual development), every 6 months for children aged 2–5 years, annually for children 5–10 years of age, and thereafter, to be decided on an individual basis (depending on the presenting ocular status). Examination may include an orthoptic examination including dynamic retinoscopy to determine the accommodative ability, anterior and posterior segment examination, and, at an older age, corneal topography assessment [8] (if patient compliance is sufficient). The widespread application of a systematic screening and examination schedule will have an important impact on the visual development and, therefore, quality of life of patients with trisomy 21.

## Figures and Tables

**Table 1:** Summary of publications of patients with trisomy 21.

	Stirn Kranjc [2]	Ljubic et al. [4]	Akinci et al. [3]	Fimiani et al. [5]	Berk et al. [6]	da Cunha and De Castro Moreira [7]	Caputo et al. [8]	Tomita et al. [9]	Liza-Sharmini et al. [11]	Schneier et al. [12]	Jaeger [13]	Kim et al. [10]	Our study
Number of patients (age in years)	65 (0.2–13)	170 (1–34)	77 (1–17)	157 (0.1–64)	55 (NA)	152 (0.16–18)	187 (NA)	304 (7.4)	60 (1–34)	793 (1–17)	75 (15–64)	123 (0.5–14)	52 (0.2–35)
Hyperopia (%)	36.9	NA	62.3	59	52.7	26	20.9	69.1	25	62.3	13	28	26
Astigmatism (%)	29.2	72.4	59.7	28	12.7	60	22	58.5	8.3	59.7	NA	31	54
Myopia (%)	24.6	NA	7.8	9	12.7	13	22.5	NA	29.2	7.8	14	25	15
Strabismus (%)	26.1	26.5	32.5	36	21.8	38	57	36.5	26.7	32.5	41.3	25	38
Congenital cataract (%)	12.3	NA	5.1	11	20	13	NA	10.5	13.3	5.1	NA	13	5
Retinal anomalies (%)	32.2	NA	NA	6	38.1	28	NA	NA	1.7	NA	NA	15	9
Optic nerve anomalies (%)	NA	NA	NA	NA	NA	NA	NA	1.6	NA	3	NA	NA	NA

**Table 2:** Demographic table of our chart review.

	Number of patients (age in years)	Referral by a pediatrician in %	Referral by a primary-care physician in %	Referral by an ophthalmologist	Refractive disorders in %	Patients with astimgatism in %	Patients with hyperopia in %	Patients with myopia in %	Patients with nystagmus in %	Patients with strabismus in %	Patients with keratoconus in %	Patients with cataracts in %	Spectacles prescribed in %
Our study	52 (0.2–35)	60	27	13	72	54	26	15	19	38	5	6	54

**Table 3:** Survey questions and response options.

What is your occupation?	Ophthalmology resident/nonophthalmological resident/board-certified ophthalmologist/orthoptist
How long have you been practicing?	<5 years/5–10 years/>10 years
What is your field of practice?	Ophthalmologist without subspecialty/pediatric ophthalmology/retina/anterior segment/neuroophthalmology/glaucoma/oculoplastics
Where do you work?	Private practice/secondary-care center/tertiary-care center
Please select which country you are from?	Switzerland/other European countries
Do you have patients with Down syndrome?	Yes/no
How often do you treat patients with Down syndrome?	1-2×, 3–5×, 5–10×, >10×, >20×/month
At what age in average do you see your patients with Down syndrome for the first time? (open answer)	(open answer)
Do you treat patients with Down syndrome that are older than 18?	Yes/no
Do you use a screening protocol for patients with Down syndrome without ocular symptoms or complaints?	Yes/no/sometimes/I would like to, but I do not know of any screening protocols
Do you feel that children with Down syndrome receive good ophthalmological care in your country?	Yes/no
Do you refer patients with Down syndrome to a low-vision specialist?	Yes, always/yes, regularly/yes, rarely/no
Have you prescribed glasses with near addition in patients with Down syndrome?	Yes/no
What difficulties do you encounter in patients with Down syndrome? (open question)	(open question)

## Data Availability

The data used in this study are included in the supplementary materials.

## Supplementary Materials

Supplementary Materials Supplementary materials include the details of the survey and its results and a summarized table of the most common ophthalmic anomalies/pathologies in patients with trisomy 21 according to different studies, as well as a demographic table of our chart review.
