# Supplementary material for: Challenges in Patients with Trisomy 21: A Review of Current Knowledge and Recommendations
Source: J Ophthalmol. 2021 May 26;2021:8870680. doi: 10.1155/2021/8870680 (PMC8172292; doi:10.1155/2021/8870680)
Supplement: Supplementary Materials — include the details of the survey and its results and a summarized table of the most common ophthalmic anomalies/pathologies in patients with trisomy 21 according to different studies, as well as a demographic table of our chart review. [file 8870680.f1.docx]

**Supplementary material**

Table S1

| What is your occupation? | Ophthalmology resident: 25%  Non-ophthalmology resident: 1%,  Board certified ophthalmologist: 74%  Orthoptist: 0% |
| --- | --- |
| How long have you been practicing? | <5 years: 15%  5-10 years: 11%  > 10 years:74% |
| What is your field of practice? (multiple answers possible) | Ophthalmologist without subspecialty: 44%  Pediatric ophthalmology: 37%  Retina: 9%  Anterior segment: 22%  Neuroophthalmology: 9%  Glaucoma:10%  Oculoplastics: 7% |
| Where do you work? | Private practice: 49%  Secondary care center: 23%  Tertiary care center: 28% |
| Please select which country you are from | Switzerland: 41%  Other European country: 59% |
| Do you have patients with Down syndrome? | Yes: 89%  No: 11% |
| How often do you treat patients with Down syndrome? | 1- 2x/ month: 78%  3 - 5x/ month: 9%  5 - 10x/ month: 8%  > 10x/ month: 5%  > 20x / month: 0% |
| At what age in average do you see your patients with Down syndrome for the first time? (open answer) | Variable: from birth- 45 years of age |
| Do you treat patients with Down syndrome that are older than 18? | Yes: 77%  No: 23% |
| Do you use a screening protocol for patients with Down syndrome WITHOUT ocular symptoms or complaints? | Yes: 26%  No: 45%  Sometimes: 4%  I would like to, but I don't know of any screening protocols: 25% |
| Do you feel that children with Down syndrome receive good ophthalmological care in your country? | Yes: 85%  No: 15% |
| Do you refer patients with Down syndrome to a low vision specialist? | Yes, always: 7%  Yes, regularly: 17%  Yes, rarely: 45%  No: 31% |
| Have you prescribed glasses with near addition in patients with Down syndrome? | Yes: 62%  No: 39% |
| What difficulties do you encounter in patients with Down syndrome? (open question) | See result section |

Table 1: Summary of publications of patients with trisomy 21

|  | Stirn Kranjc 2012^2^ | Ljubic et al. 2011^3^ | Akinci et al. 2009^4^ | Fimiani et al. 2007^5^ | Berk et al. 1996^6^ | Da Cunha et al. 1996^7^ | Caputo et al. 1989^8^ | Tomita et al. 2013^9^ | Liza-Sharmini et al. 2006^10^ | Schneier et al. 2013^11^ | Jaeger 1980^12^ | Kim et al. 2002^13^ | Our study |
| --- | --- | --- | --- | --- | --- | --- | --- | --- | --- | --- | --- | --- | --- |
| Number of patients  (age in years) | 65  (0.2-13) | 170  (1-34) | 77  (1-17) | 157  (0.1-64) | 55 (NA) | 152 (0.16-18) | 187  (NA) | 304  (7.4) | 60  (1-34) | 793  (1-17) | 75  (15- 64) | 123  (0.5-14) | 52  (0.2-35) |
| Hyperopia (%) | 36.9 | NA | 62.3 | 59 | 52.7 | 26 | 20.9 | 69.1 | 25 | 62.3 | 13 | 28 | 26 |
| Astigmatism (%) | 29.2 | 72.4 | 59.7 | 28 | 12.7 | 60 | 22 | 58.5 | 8.3 | 59.7 | NA | 31 | 54 |
| Myopia (%) | 24.6 | NA | 7.8 | 9 | 12.7 | 13 | 22.5 | NA | 29.2 | 7.8 | 14 | 25 | 15 |
| Strabismus (%) | 26.1 | 26.5 | 32.5 | 36 | 21.8 | 38 | 57 | 36.5 | 26.7 | 32.5 | 41.3 | 25 | 38 |
| Congenital cataract (%) | 12.3 | NA | 5.1 | 11 | 20 | 13 | NA | 10.5 | 13.3 | 5.1 | NA | 13 | 5 |
| Retinal anomalies (%) | 32.2 | NA | NA | 6 | 38.1 | 28 | NA | NA | 1.7 | NA | NA | 15 | 9 |
| Optic nerve anomalies (%) | NA | NA | NA | NA | NA | NA | NA | 1.6 | NA | 3 | NA | NA | NA |

Table 2: Survey questions and response options

| What is your occupation? | Ophthalmology resident/  Non-ophthalmological resident/  Board certified ophthalmologist  Orthoptist |
| --- | --- |
| How long have you been practicing? | <5 years/ 5-10 years/ > 10 years |
| What is your field of practice? | Ophthalmologist without subspecialty/ Pediatric ophthalmology/ Retina/ Anterior segment/ Neuroophthalmology/  Glaucoma/ Oculoplastics |
| Where do you work? | Private practice/ Secondary care center/ Tertiary care center |
| Please select which country you are from | Switzerland/ other European country |
| Do you have patients with Down syndrome? | Yes/ No |
| How often do you treat patients with Down syndrome? | 1- 2x, 3 - 5x, 5 - 10x, > 10x, > 20x / month |
| At what age in average do you see your patients with Down syndrome for the first time? (open answer) | (Open answer) |
| Do you treat patients with Down syndrome that are older than 18? | Yes/ No |
| Do you use a screening protocol for patients with Down syndrome WITHOUT ocular symptoms or complaints? | Yes / No / Sometimes / I would like to, but I don't know of any screening protocols |
| Do you feel that children with Down syndrome receive good ophthalmological care in your country? | Yes/ No |
| Do you refer patients with Down syndrome to a low vision specialist? | Yes, always/ Yes, regularly/ Yes, rarely/ No |
| Have you prescribed glasses with near addition in patients with Down syndrome? | Yes/ No |
| What difficulties do you encounter in patients with Down syndrome? (open question) | (Open Question) |

Table 3: Demographic table of our chart review

|  | Number of patients  (age in years) | Referral by pediatrician  In % | Referral by primary care physician in % | Referral by ophthalmologist | Refractive disorders in % | Patients with Astimgatism in % | Patients with hyperopia in % | Patients with myopia in % | Patients with nystagmus in % | Patients with strabismus in % | Patients with keratoconus in % | Patients with cataracts in % | Spectacles prescribed in % |
| --- | --- | --- | --- | --- | --- | --- | --- | --- | --- | --- | --- | --- | --- |
| Our Study | 52  (0.2-35) | 60 | 27 | 13 | 72 | 54 | 26 | 15 | 19 | 38 | 5 | 6 | 54 |
